# The presence of abnormal palpatory findings in the sacrococcygeal area is correlated with chronic pelvic pain: a cross-sectional study

**DOI:** 10.1007/s11255-025-04521-2

**Published:** 2025-04-25

**Authors:** Daniele Origo, Fulvio Dal Farra, Marco Tramontano

**Affiliations:** 1 Research Department, SOMA Osteopathic Institute Milan, 20126 Milan, Italy; 2 Department of Information Engineering, University of Brescia, 25123 Brescia, Italy; 3 Department of Biomedical and Neuromotor Sciences, University of Bologna, 40138 Bologna, Italy; 4 Unit of Occupational Medicine, IRCCS Azienda Ospedaliero-Universitaria Di Bologna, 40138 Bologna, Italy

**Keywords:** Chronic pelvic pain, Chronic pelvic pain syndrome, Coccydynia, Pelvic floor, Sacrococcygeal, Manual therapy, Palpatory findings

## Abstract

**Objective:**

This study examines the prevalence of abnormal palpatory findings (APFs) in the different pelvic areas among individuals with chronic pelvic pain syndrome (CPP-CPPS) and assesses correlations between APFs and clinical and psychosocial symptoms.

**Methods:**

In this cross-sectional study, 326 participants (162 CPP-CPPS patients, 164 controls) underwent a standardized palpatory assessment of the sacroiliac, sacrococcygeal, and pelvic floor regions. The manual procedure was performed by two expert physiotherapists with a certification in osteopathic manipulation, following a consensus training. We assessed symptom severity and psychosocial variables using the NIH Chronic Prostatitis Symptom Index (NIH-CPSI), the Hospital Anxiety and Depression Scale (HADS), and the Fear Avoidance Belief Questionnaire (FABQ). Correlation analyses explored relationships between APFs, the presence of pain, and psychosocial variables.

**Results:**

APFs were significantly associated with CPP/CPPS, particularly in the sacrococcygeal (*r* = 0.609, *p* < 0.01) and pelvic floor (*r* = 0.620, *p* < 0.01) regions, indicating a moderate-to-strong correlation. The multivariate analysis confirmed that sacrococcygeal APFs (OR 3.02, 95% CI 1.96–4.65, *p* < 0.001) and pelvic floor APFs (OR 2.99, 95% CI 1.87–4.78, *p* < 0.001) were independently associated with CPP/CPPS, whereas sacroiliac findings showed a weak correlation. The correlations between APFs and psychosocial issues (anxiety, depression, fear-avoidance) were weak (*r* = 0.25).

**Conclusions:**

Sacrococcygeal and pelvic floor APFs appear to be important clinical markers of CPP/CPPS. Their presence may help identify patients who could benefit from targeted manual therapy as part of multimodal management. Further research should evaluate the prognostic value of these findings.

## Introduction

Chronic pelvic pain (CPP) is defined as non-malignant pain perceived in structures related to the pelvis, persisting continuously or recurrently for a duration of at least 3 months, according to the European Association of Urology (EAU) guidelines. The chronic pelvic pain syndrome (CPPS) is characterized by the presence of CPP associated with urinary, gastrointestinal, or sexual symptoms without identifiable pathology, following the EAU and ICD-11 criteria [1, 2].

Non-specific diffuse pain, in the absence of identifiable pathology, may affect one or more pelvic organs, the abdominal wall, and the genital area, and can also be accompanied by systemic symptoms [3]. Moreover, various conditions characterized by central sensitization mechanisms such as irritable bowel syndrome, interstitial cystitis, painful bladder syndrome, and fibromyalgia, are highly prevalent among individuals with CPP/CPPS, highlighting the importance of addressing the patient's physical, psychological, and social needs [4, 5].

The epidemiological data on CPP/CPPS are disparate, due to the varying quality of the studies. Prevalence is definitely higher in women, with a lifetime prevalence ranging from 5.7% to 26.6% [6], while 10% of men experience CPP/CPPS [7].

Patients typically present dysfunctions in the pelvic floor muscles, which resulted in hyperactivity. Tissue palpation may reveal the presence of trigger points (TPs) in adjacent muscles, such as the piriformis, gluteus maximus, and iliopsoas [8], as well as tenderness in the suprapubic, abdominal, and intrapelvic regions [9].

Multimodal physical treatments (patient education, biofeedback, manual therapy, and injections to address abdominal and pelvic TPs) appeared to be an effective therapeutic choice [10, 11]. In a recent position statement, the Italian Society of Colo-Rectal Surgery emphasized the need for a multimodal clinical approach in CPP/CPPS, highlighting the importance of a careful assessment of the musculoskeletal system, but also posture, joints, and tissues in general, which are often underrated [12]. In this context, the physical assessment of postures, joints mobility and tissues characteristics are frequently performed by different healthcare professionals such as physiotherapists, osteopaths and chiropractors, according to their competences and educational backgrounds [13]. The presence of abnormal palpatory findings (APFs) in terms of joint mobility restriction, alterations of tissue texture and tenderness areas are detectable by palpation procedures and guides the physical assessment of such approaches [14].

Although the contribution of the myofascial dysfunctions has been largely demonstrated in CPP/CPPS, so far no study investigated which parts of the pelvic region appear to be the most involved in this condition. From this perspective, it becomes relevant to take into account the anatomical and functional relationships of the pelvic floor (e.g., muscles, ligaments, and fascia, forming a sort of sling supporting the internal organs). These soft tissues attach to the pelvic girdle and posteriorly to the sacrum and coccyx, which are crucial for anchoring the muscles forming the pelvic floor [15].

Our hypothesis is that specific pelvic dysfunctional areas, detectable in a form of APFs, might be more involved than others in the contribution to chronic pain.

Therefore, the primary aim of this study is to assess and quantify the presence of abnormal palpatory findings (APFs) of the pelvic area in patients with CPPS. Secondarily, to correlate the presence of APFs to symptoms, signs, physical and psychosocial variables of people presenting CPP/CPPS.

## Methods

### Study design and setting

This cross-sectional investigation was carried out between September 2023 and June 2024. All the procedures have been conducted in a rehabilitative outpatient clinic located in Milan, Italy. The study protocol was approved by the Institutional Review Board of the SOMA Clinical Institute (registration number: SIOM-AA0025), and all the participants gave informed consent before being tested. This study was conceived and reported following the “Strengthening the reporting of observational studies in epidemiology (STROBE) statement” checklist for cross-sectional studies [16].

### Participants

The individuals with CPP/CPPS were recruited on a voluntary basis from the population of patients referring to the clinic. Each patient who had already received a medical diagnosis of CPP/CPPS was offered the possibility to participate. Eligible participants were individuals of any gender, aged 18 to 65 years, who had received a documented diagnosis of chronic pelvic pain syndrome (CPP/CPPS) by their referring physicians, based on appropriate clinical and instrumental assessments ensuring the exclusion of alternative specific diagnoses (e.g., endometriosis, pelvic inflammatory disease, neuropathies). The people without CPP/CPPS were recruited by flyers among students and personnel attending the clinic, or the annex college campus.

Inclusion criteria were people aged 18–65, with or without CPP/CPPS. Conversely, we excluded individuals with serious musculoskeletal disorders (e.g., multiple fractures, rheumatism), unresolved cancer, current pregnancy or any impossibility to undergo a palpatory assessment in the pelvic area, both due to physical or cognitive issues. In addition, we also excluded people receiving physical or manipulative treatments elsewhere. Each participant with CPP/CPPS was matched to a subject without pain, based on their age and gender.

### Outcome measures

All the participants recruited for the study received a booklet requesting socio-demographics, clinical information, and containing three different questionnaires.

This booklet included questions related to symptoms localization and duration, physical activity (frequency, duration, and intensity) and previous trauma. In addition, the following questionnaires were incorporated.

The “NIH-Chronic Prostatitis Symptom Index” (NIH-CPSI) was implemented to investigate the symptom severity; it contains three main domains: pain, urinary symptoms, and quality of life impact. The NIH-CPSI is considered a reliable and cross-culturally validated instrument, frequently considered in the functional assessment of people with CPP/CPPS [17].

The “Hospital Anxiety and Depression Scale” (HADS) is a 14-items questionnaire based on a 4-points Likert score (0–3), and it is considered a reliable instrument to detect anxiety and depression states, measured by two distinct scores [18].

The “Fear Avoidance Belief Questionnaire” (FABQ) measures fear of pain and consequent activity avoidance, and it has been successfully translated into Italian language. It is composed of 16 items, based on a 7-points Likert scale (0–6) expressing the agreement the subject has with regards to the different statements [19].

### Physical assessment

Subsequently, each subject underwent a standardized palpatory assessment performed by two expert physiotherapists (more than 15 years of experience), with a certification in osteopathic manipulation. Before testing, the assessors went through a procedure of consensus training to ensure the highest concordance between the two operators. Such a standardized training consisted of 9 h of practice, divided into three main sessions (3 h each), and a 2-h final meeting when consensus has been definitely reached. With regard to this, the use of a Digital Hand-Held Dynamometer (PainTest™ FPX 25 Algometer, Wagner Instruments, Greenwich, USA) was used, since it proved to be a valid and reliable tool [20]. The assessors agreed upon a palpatory force ranging between 0.80 and 1 kg, and after adequate training, in 95% of the trials, they were able to consistently maintain this force range. The consensus procedure concluded successfully, as the first 20 participants (10 for each group) were assessed by both trained operators, showing good inter-rater agreement for both dysfunction detection (K = 0.73) and grading (weighted K = 0.62). All the other individuals included in the study were only assessed by one physiotherapist.

The manual assessment was exclusively aimed to detect APFs in the ileo-sacral joints, sacrococcygeal region and in the pelvic floor area. Following the TART model principles, alterations in the tissue texture, joint movement reduction and tenderness or pain evocation are considerable APFs, attributable to the presence of somatic dysfunction. The TART framework (T: texture; A: asymmetry; R: restriction; T: tenderness) is a procedure widely used by manual therapists to detect the presence of somatic dysfunctions. In recent years, new research has been conducted to assess the validity and reliability of this method [21, 22]. From this point on, the expressions “APFs” and “dysfunction” are considered synonymous.

The procedure consisted of three different standardized maneuvers, aimed to assess the presence and the grade of dysfunction of two joint complexes and a soft-tissue region.

**Fig. 1:**
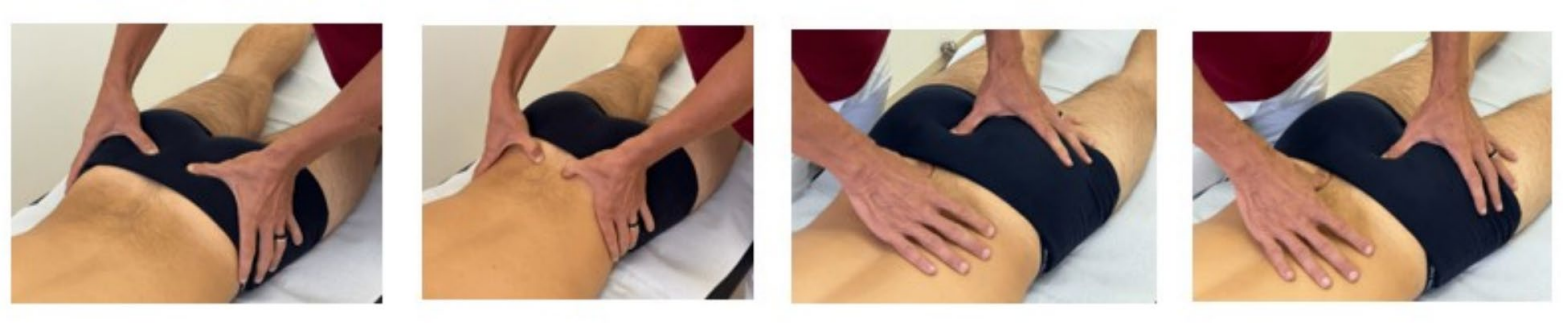
Sacroiliac joint assessment

The first technique was oriented to the sacroiliac joint with the patient in a prone position, the operators placed two fingers on the inferior groove of the posterior inferior iliac spine, to verify the position in the frontal plane. The fingers are then flexed approximately 30° medially and caudally to contact the sacral base, which is assessed in the coronal plane. The fingers are subsequently moved to the inferior lateral angle of the sacrum to perform a similar assessment in the coronal plane. Next, the operators placed the fingers on the sacral base and the inferior lateral angle of the sacrum ipsilaterally, applying pressure to evaluate the ability of the sacrum to perform nutation and counternutation, as well as to assess for palpatory tenderness. The assessors then placed the fingers on the left base and the right inferior lateral angle of the sacrum, then vice versa, applying pressure to induce movement, assessing for both the range of motion and tenderness [23] (Fig. 1).

**Fig. 2:**
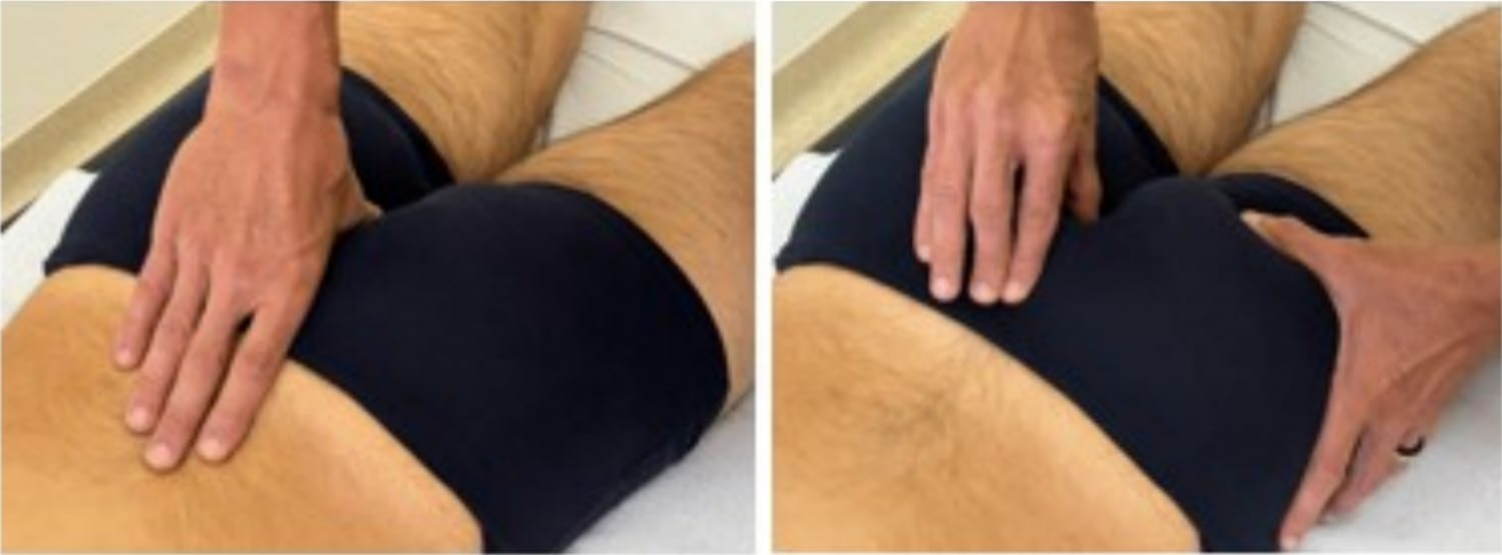
Sacrococcygeal joint assessment

The second technique was oriented to the sacrococcygeal joint assessment: the assessors placed a finger on the coccyx with the patient in a prone position, palpating the entire contour of the bone, while asking the patient the presence of pain. The patient was then instructed to take a deep breath, and operators observed the movement of the coccyx facilitated by the pelvic diaphragm muscles, which follow the downward movement of the thoracic diaphragm during in-breathing [24] (Fig. 2).

**Fig. 3:**
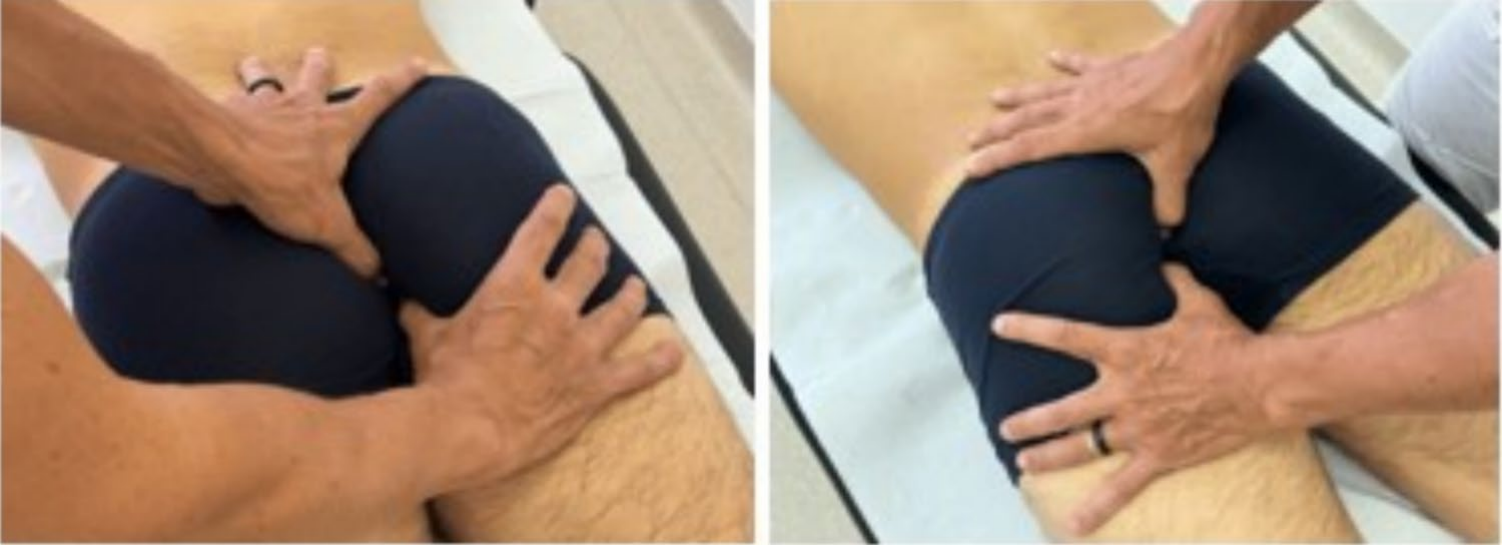
Pelvic floor assessment

Regarding the third technique: the assessors applied pressure to the pelvic diaphragm muscles between the ischium, pubic bone, and coccyx, evaluating for any palpatory tenderness and the ability of the muscles to move caudally in conjunction with a deep inspiration [25] (Fig. 3).

Finally, the assessors were asked to grade the dysfunction for each of the assessed anatomical areas using the following criteria: a score of 0 indicated the absence of any APFs; a score of 1 was assigned when a single APF was present (such as movement restriction, altered tissue texture, or pain on palpation); a score of 2 was given when two or more APFs were simultaneously present.

### Sample size calculation

In this study, no study sample calculation was performed, since the presence of physical dysfunctions (joint restrictions, tissue texture alterations, tenderness) were not measured in any previous study dealing with CPP/CPPS, and no expected mean for the outcome of interest was retrievable in literature.

For these reasons, the consecutive enrolling of the subjects stopped when a considerable number of participants was definitely reached for each group.

### Statistical analysis

The main characteristics of the sample were reported in a descriptive way.

The distribution for continuous variables was tested via Kolmogorov–Smirnov test. Since no normal distribution was found, the U-Mann–Whitney test for independent samples was applied to test any possible differences between the two groups in terms of age, thus verifying the success of the matching procedure.

Spearman correlation test was implemented to verify any possible correlation between the condition of CPP/CPPS and all the other variables of interest, such as the presence and severity of pelvic dysfunctions, the questionnaire scores, levels of physical activity and a history of a previous trauma. As a secondary analysis, a multivariate analysis (logistic regression) was considered to highlight any possible association between the presence of specific APFs (independent variables or covariates) and the condition of CPP/CPPS (dependent variable).

Statistical analyses were performed using IBM SPSS Statistics for Windows, Version 23.0 (IBM Corp., Armonk, NY, USA).

## Results

### Characteristics of the sample

A total of 326 individuals were recruited, specifically 162 people with a CPP/CPPS condition and 164 healthy people. The mean age of the participants was 35.62 ± 12.2, and all the main characteristics of the sample are detailed in Table 1.

**Table 1:** Characteristics of the sample

	CPP/CPPS	Healthy people
Number per group	162	164
Gender (M/F)	59/103	59/105
Age (IQR)	32.5 (26–43)	31.5 (26–45)
Physical activity (no. of time/week)	1 (0–3)	2 (0–3)
Physical activity (no. of hours/week)	2 (0–4)	3 (0–6)
Sacro-coccygeal dysfunction (grading 0–2)	2 (1–2)	0 (0–0)
Sacro-iliac dysfunction (grading 0–2)	1 (0–1)	0 (0–1)
Pelvic floor dysfunction (grading 0–2)	2 (1–2)	0 (0–0)
NIH-CPSI pain (IQR)	8 (5–11)	0 (0–1)
NIH-CPSI urinary (IQR)	2 (0–5)	1 (0–2)
NIH-CPSI QoL (IQR)	7 (4–9)	0 (0–2)
NIH-CPSI total score (IQR)	17 (10–22)	2 (0–7)
HADS anxiety (IQR)	8 (5–12)	6 (4–9)
HADS depression (IQR)	7 (4–10)	5 (2–8)
HADS total score (IQR)	15 (9–21)	11 (6–16)
FABQ score (IQR)	19 (6–31)	6 (0–17)

CPP/CPPS, chronic pelvic pain/chronic pelvic pain syndrome; IQR, interquartile range; NIH-CPSI, National Institute of Health-Chronic Prostatitis Symptoms Index; HADS, Hospital Anxiety and Depression Scale; FABQ, Fear Avoidance Belief Questionnaire

The majority of people with CPP/CPPS presented symptoms for more than 3 years (38.9%) and only the 8% of the cases complained of symptoms for 3–6 months. In addition, 15.4% of the cases reported sexual dysfunctions, the 6.8% complained of urinary disturbances and 59.9% reported both of the functions as impaired.

As for the localization, 41% of CPP/CPPS people reported symptoms in different abdominal and pelvic areas, 13% in the pubic region, 9.9% in the coccygeal area and the 6.2% in the perineal region. Further details are reported in Table 2.

**Table 2:** Symptoms’ characteristics in the CPP/CPPS group

	Num. of individuals (%)
Symptoms functional impact	
None	29 (18%)
Sexual	25 (15%)
Urinary	11 (7%)
Both	97 (60%)
Symptoms duration	
3–6 months	13 (8%)
6–12 months	34 (21%)
1–3 years	52 (32%)
> 3 years	63 (39%)
Symptoms localization	
No specific localization	25 (16%)
Lumbo-Sacral	4 (3%)
Sacro-coccygeal	16 (10%)
Lower abdomen	8 (5%)
Sacro-iliac	7 (4%)
Pubis	21 (14%)
Perineum	10 (7%)
More than one area	63 (41%)

APFs in the sacrococcygeal joint were present in the 78% of the people suffering from CPP/CPPS, the sacro-iliac APFs in the 72%, although 56% of these cases were classified as of “grade 1”. The APFs of the pelvic floor was detected in 80% of the people with CPP/CPPS.

### Correlations analysis

A significant moderate-to-strong correlation was found between the presence of CPP/CPPS and sacrococcygeal and pelvic floor APFs (*r* = 0.609 and *r* = 0.620, *p* < 0.01, respectively), but not with the sacroiliac dysfunction where the correlation was present, yet weak (*r* = 0.269, *p* < 0.01). The correlation between the presence of CPP/CPPS with the severity of the APFs overlaps the above reported data (*r* = 0.655, *r* = 0.642, *r* = 0.315, *p* < 0.01, respectively).

A significant weak correlation was found between having CPP/CPPS and the HADS total score (*r* = 0.264, *p* < 0.01), also considering both the anxiety (*r* = 0.258, *p* < 0.01) and the depression sub-scores (*r* = 0.210, *p* < 0.01). In the same way, a significant weak correlation was detected with the FABQ score (*r* = 0.273, *p* < 0.01). Finally, a minimal significant correlation was found between presenting a CPP/CPPS condition and the frequency or the amount of physical activity (*r* = 0.126 and *r* = 0.140, *p* < 0.05 respectively).

All the correlations are reported in Table 3.

**Table 3:** Correlation matrix for the investigated variables

	CPP/CPPS	PA dur	PA t/w	PA h/w	Tr y/n	SC y/n	SC sev	SI y/n	SI sev	PF y/n	PF sev	HADS anx	HADS dep	HADS tot	FABQ
CPP/CPPS	1														
PA dur	− 0.055	1													
PA t/w	− 0.126	0.686	1												
PA h/w	− 0.14	0.694	0.945	1											
Tr y/n	− 0.161	− 0.111	− 0.042	− 0.051	1										
SC y/n	0.609	− 0.042	− 0.089	− 0.101	− 0.143	1									
SC sev	0.655	− 0.035	− 0.094	− 0.104	− 0.151	0.965	1								
SI y/n	0.269	− 0.025	0.01	− 0.012	− 0.105	0.325	0.303	1							
SI sev	0.315	− 0.033	− 0.004	− 0.026	− 0.11	0.357	0.336	0.946	1						
PF y7n	0.62	− 0.017	− 0.119	− 0.139	− 0.073	0.638	0.696	0.213	0.277	1					
PF sev	0.642	− 0.005	− 0.097	− 0.108	− 0.102	0.675	0.75	0.239	0.295	0.948	1				
HADS anx	0.258	− 0.136	− 0.13	− 0.14	− 0.045	0.312	0.318	0.158	0.151	0.249	0.264	1			
HADS dep	0.21	− 0.179	− 0.19	− 0.192	− 0.037	0.242	0.252	0.12	0.43	0.198	0.219	0.604	1		
HADS Tot	0.264	− 0.171	− 0.178	− 0.185	− 0.044	0.314	0.322	0.101	0.116	0.256	0.277	0.884	0.899	1	
FABQ tot	0.273	− 0.123	− 0.181	− 0.17	− 0.125	0.218	0.228	0.175	0.173	0.27	0.282	0.228	0.283	0.284	1

CPP/CPPS, chronic pelvic pain/chronic pelvic pain syndrome; PA dur, physical activity duration; PA t/w, physical activity times/week; PA h/w, physical activity hours/week; Tr, trauma; SC Y/n, sacrococcygeal yes/no; SC/sev, sacrococcygeal severity; SI, sacro-iliac; PF, pelvic floor; HADS anx, HADS anxiety; HADS dep, HADS depression

### Secondary analysis

A multivariate analysis was implemented to provide a prediction model considering the presence of CPP/CPPS as a dependent variable and the APFs as covariates. The sacro-coccygeal and the pelvic floor APFs’ severity resulted as factors significantly associated with CPP/CPPS [exp B: 3.02 (1.96–4.65) and 2.99 (1.87–4.78), *p* < 0.001, respectively). No significant association was found with the sacroiliac dysfunction [exp B: 1.57 (0.97–2.53), *p* = 0.061. Further details are reported in Table 4.

**Table 4:** Logistic regression analysis (multivariate analysis)

Dysfunction	B	Exp(B)	95% CI
Sacro-coccygeal dysfunction	1.1*	3.02	(1.96–4.65)
Sacro-iliac dysfunction	0.45	1.57	(0.97–2.53)
Pelvic floor dysfunction	1.09*	2.99	(1.87–4.78)

Dependent variable: presence of a CPP/CPPS condition. Covariates: sacrococcygeal dysfunction severity; sacroiliac dysfunction severity; pelvic floor dysfunction. CI: confidence interval

*p < 0.001

## Discussion

### Summary of results

To the best of our knowledge, this study represents the first attempt to understand whether APFs are prevalent in the sacrococcygeal, sacroiliac, or pelvic floor areas in patients with CPP/CPPS. Our results indicate a significant presence of these clinical signs at the level of the coccyx and the pelvic muscles, which are closely related from an anatomical and functional point of view [15]. Furthermore, the distribution and the impact of the symptoms in our sample resulted in line with previous epidemiological studies [5, 6]. Indeed, pain appears to be located in various pelvic areas, both urological and sexual disturbances are highly prevalent in this population and the impact of this condition largely affects people in terms of perceived pain, urinary function and quality of life, as reported by NIH-CPSI questionnaire. Weak correlations were detected between the presence of pelvic APFs and levels of anxiety, depression and fear-avoidance beliefs, whereas no significant correlations were obtained between the presence of APFs and previous trauma or the frequency, duration and volume of physical activity.

### Clinical interpretation

The findings of this study are consistent with existing literature regarding the contribution of pelvic myofascial and articular structures in subgroups of patients suffering from CPP/CPPS [26]. It is noteworthy to highlight the hypothesis that myofascial release, through a multimodal approach, could restore the physiological blood flow necessary for patient comfort, as a reduction in blood flow to the tissues may sustain this debilitating syndrome [27]. Consequently, pelvic muscle dysfunction and their anatomical relationships, combined with psychosocial factors and pain misperception, are involved variables and a timely understanding of these factors is crucial for an optimal therapeutic approach [28].

Although in CPP/CPPS the scientific literature rightly considered traumatic experiences, distress, pain and non-pelvic comorbidities [29], the results of our study reveal only a weak correlation with the anxiety and depression scale, and with the fear-avoidance beliefs questionnaire. This fact raises the question on whether the emotional state is the cause or the effect of a condition that so significantly impacts quality of life. It seems easy to imagine how such a condition could induce neurobiological mechanisms related to anxiety, depression and distress [30].

Our results did not show any relevant correlation, nor a statistically significant association between sacro-iliac joint APFs and the presence of CPP/CPPS. The sacroiliac joint plays a key role in distributing forces from the upper body, and its movement and load-bearing capacity are influenced by the lumbosacral spine [31]. It is involved in pelvic girdle pain in cases of pelvic bone asymmetry, movement asymmetry, leg length discrepancy, joint instability, pregnancies, inflammation, fractures, or the presence of tender or trigger points in ligaments and muscles, as well as altered muscular recruitment [32, 33]. In summary, the SIJ seems to be linked to anatomical, biomechanical, and functional lumbopelvic kinematic conditions, which are not correlated with the specific clinical condition and symptomatology of CPP/CPPS.

The high prevalence of sacrococcygeal APFs in CPP/CPPS warrants further clinical consideration. This joint is included in the taxonomy of CPP/CPPS, both from the perspective of symptomatic manifestation and in terms of assessment opportunities [34]. The coccyx serves as a key attachment point for several pelvic floor muscles key pelvic floor muscles and ligaments [35], which may help explain why dysfunction in this region can contribute to increased pelvic floor tension and pain. Given the high prevalence of sacrococcygeal APFs in individuals with CPP/CPPS, such findings may act as useful clinical markers for identifying patients who could benefit from targeted interventions. In clinical practice, the recognition of sacrococcygeal and pelvic floor dysfunctions during examination may facilitate patient stratification into subgroups more likely to respond to focused manual therapy or other region-specific treatments.

Women with coccydynia report an increase in pain during the premenstrual period, and sometimes dyspareunia. The character of the pain appears to be more related to spasm of the levator ani muscle, as patients complain of pain during defecation or sexual intercourse [36]. The levator ani muscle, which inserts onto the coccyx, can be hypertonic, with palpable trigger points, and manifests with proctalgia, pelvic muscle tension, and ano-vaginal pain [37].

In this context, clinical and radiological evaluation of the coccyx is useful to assess its shape, joint physiology, palpatory sensitivity, and tissue quality [38]. Prolonged sedentary positions can be a dysfunctional cause of the coccyx, with Shapiro attributing it to prolonged incorrect sitting positions (slouched position), akin to a “television disease” [39]. Indeed, the importance of altered sitting postures, associated with fascial insults in the abdominal and pelvic areas (e.g., repeated inflammations, surgical scars), has recently been emphasized as an exposure factor in the development of CPP/CPPS [40, 41].

Both coccydynia and pelvic pain syndrome are associated with pelvic floor dysfunctions, with signs and symptoms that may overlap in some cases, even if they are not classified as diseases [42]. This may lead to diagnostic delays, the development of chronic pain with the associated psychosocial consequences, which can dramatically evolve [43].

Nonetheless, the contractures may have a functional origin related to daily activities, or they may have a systemic, metabolic, or endocrinological origin (such as thyroid and parathyroid dysfunctions), or result from venous insufficiency [44]. Therefore, it is important to educate the patient about correct postural behaviors and the contribution of minor trauma and inflammation to symptom expression, to engage the patient in the treatment process.

In summary, pain is the defining feature of CPP/CPPS condition, rather than the consequence of underlying pathological processes. In other words, pain itself constitutes the pathological process [5]. Coccygodynia is considered a symptom rather than a distinct clinical disease, thus not requiring a separate clinical recognition [45]. Muscular hypertonicity can be clinically identified, even if the manual assessment lacks high reliability [22]. These factors highlight the need for a high clinical expertise in the assessment and management of CPP/CPPS [2].

It is worth emphasizing that APFs may be present in patients with CPP/CPPS, regardless of pain topography. For this reason, they could be useful in sub-grouping patients who may potentially respond to manual therapy, as well as in identifying the likelihood for developing CPP/CPPS.

Future research, through longitudinal studies, could verify whether coccyx and pelvic muscle dysfunctions can be considered clinical predictors for the development of CPP/CPPS.

### Generalizability of the results

Considering the sample size and the selection criteria we adopted for this study, we have reason to assume that our sample could be representative of the real population. However, the totality of the recruited subjects attended the same clinical setting, and the vast majority of healthy people were students or employers of the Institute, so that a selection bias might be present.

The findings we obtained were related to the presence of APFs, which can be detected through a palpatory assessment. As largely known, manual procedures are prone to reliability issues, which can affect both internal and external validity of a study [22, 46]. However, studies demonstrated that procedures of consensus training and standardization of the manual techniques may improve intra- and inter-operator agreements. With regard to this, our experience is consistent with the results of these studies, since our operators agreed in 95% of the observations during the consensus stage. In addition, we reported a detailed description of the manual techniques we implemented for this study, to improve the generalization of this study.

Palpation represents a valuable tool in the clinical assessment of pelvic musculoskeletal disorders. These components contribute to a comprehensive clinical picture when dealing with complex syndromes such as CPP/CPPS. Although the reliability of such techniques may vary depending on operator expertise, standardization, and the anatomical region explored, recent efforts in training and procedural consensus—such as the one employed in our study—can enhance both inter-rater agreement and clinical applicability. Incorporating these elements into a broader examination protocol helps clinicians better identify dysfunctions that may be addressed through specific therapeutic interventions. Furthermore, it is important to remark how these types of procedures are safe, easy-practicable and cost-effective, so that they are reproducible in different conditions and in various clinical settings [47].

### Limitations

The limitations of this study include the intrinsic constraints of a cross-sectional design, which prevents the establishment of a causal relationship between the presence of sacrococcygeal and pelvic floor APFs, and the development of CPP/CPPS. Additionally, the absence of an a priori sample size calculation may have limited the study’s statistical power, potentially affecting our ability to detect smaller effects and influencing the interpretation and generalizability of the findings. Potential selection bias cannot be excluded, as participants were drawn from a single clinical setting. Furthermore, although both examiners underwent extensive consensus training to standardize the palpatory assessment, and inter-rater agreement on a small subset of the sample yielded satisfactory results, formal assessment of inter-rater reliability for the manual procedures was not conducted. This limitation may introduce variability in the findings and potentially affect the consistency and generalizability of our results.

## Conclusions

This study highlights a statistically significant correlation between sacrococcygeal and pelvic muscle APFs, and the presence of CPP/CPPS. While the cross-sectional design precludes the evidence of causality, our findings suggest that APFs in these anatomical areas could represent relevant clinical markers for identifying patients who may benefit from targeted therapeutic interventions, such as manual therapy. Further longitudinal studies are warranted to explore the long-term impact of these dysfunctions, and to better understand their role in the development and management of CPP/CPPS.

## Data Availability

Data is provided within the manuscript or supplementary information files.
